# Anaesthesiologist-led Multidisciplinary Model for Evaluating High-risk Obstructive Sleep Apnea (OSA) Surgical Patients

**DOI:** 10.37825/2239-9747.1062

**Published:** 2024-10-07

**Authors:** Giuseppe Mincolelli, Matteo L. Leoni, Marco Cascella, Marco Mercieri, Ruggero M. Corso

**Affiliations:** a UOC of Anesthesia and Resuscitation II, Fondazione IRCCS Casa Sollievo Della Sofferenza, San Giovanni Rotondo, 71013, Foggia, Italy; b Department of Medical and Surgical Sciences and Translational Medicine, Sapienza University of Rome, Rome, Italy; c Department of Medicine, Surgery and Dentistry, University of Salerno, 84081, Baronissi, Italy; d Department of Anesthesiology and Intensive Care, Guglielmo da Saliceto Hospital, 29121 Piacenza, Italy

**Keywords:** Obstructive sleep apnea (OSA), Drug-induced sleep endoscopy (DISE), Multidisciplinary approach, High-risk surgery, Patient-centered care

*Dear Editor*,

Obstructive sleep apnea (OSA) is a significant health concern with serious consequences, including excessive daytime sleepiness, cognitive disturbances, depression, hypertension, and cardiovascular and cerebrovascular diseases. High-quality surgical care for OSA is patient-centered, prioritizing the individual’s needs and involving the entire healthcare team. We believe that appropriate care goes beyond simply choosing the correct procedure for a patient’s diagnosis. Instead, it requires a comprehensive approach that considers the patient’s overall health, including any existing comorbidities, opportunities for pre-surgery improvement, a careful assessment of risks and benefits, and the potential for both short- and long-term recovery. Consequently, improving the appropriateness of surgical interventions can reduce postoperative complications, thereby lowering overall care costs [1]. The objective of this communication is to describe the development and implementation of an anaesthesiologist-led multidisciplinary committee to evaluate high-risk OSA surgical patients and enhance the appropriateness of surgical care. For patients undergoing complex or high-risk surgeries, especially those with multiple comorbidities, a collaborative approach involving the entire healthcare team is essential. This multidisciplinary strategy ensures optimal care through comprehensive preoperative assessment, medical optimization, and meticulous surgical planning. In managing OSA, valuable lessons can be learned from the “tumor board” model used in cancer centers, where specialists from various disciplines collaborate to formulate the most effective treatment strategies. A multidisciplinary team for OSA should include anaesthesiologists, surgeons, sleep specialists, pulmonologists, cardiologists, and nursing staff, all working together to assess each patient’s unique needs and tailor a personalized care plan. This approach helps identify potential risks and benefits, optimize patient outcomes, and ensure that surgical interventions are both appropriate and effective. However, while multidisciplinary meetings are common in cancer care, surgical case reviews often lack this comprehensive approach, focusing more on technical aspects rather than the broader context of the patient’s overall health [2]. The Royal College of Anaesthetists advocates for a multidisciplinary approach throughout the perioperative period [3]. Anaesthesiologists, with their expertise in all phases of perioperative care, are uniquely positioned to lead this collaborative effort [4]. Their involvement, from preoperative evaluation to postoperative critical care and pain management, is crucial for tailoring the surgical approach, optimizing outcomes, and enhancing the patient’s quality of life after surgery. This collaborative approach not only improves patient safety and surgical outcomes but also promotes patient-centered care by ensuring that treatment decisions are aligned with the patient’s unique needs and circumstances. Integrating the perspectives of various healthcare professionals enables a more comprehensive and cohesive treatment plan, addressing the full spectrum of patient care. This shift towards a team-based approach is essential for advancing healthcare quality in today’s complex medical landscape.

We propose establishing a multidisciplinary High-Risk Sleep Surgery Committee (HRSSC) at IRCCS “Casa Sollievo della Sofferenza” Hospital, Foggia, Italy, and developing a multidisciplinary-approved protocol for conducting Drug-Induced Sleep Endoscopy (DISE). The creation of the HRSSC was the result of extensive discussions, meetings, and consultations among the authors of this letter, who bring significant experience and hold prominent roles in this field within their respective institutions. Led by anaesthesiologists, the HRSSC will facilitate open discussions to evaluate high-risk surgical patients. The HRSSC will serve as a model for defining and promoting appropriate surgical care, mitigating risks, anticipating complications, and addressing challenges throughout the perioperative period. In the HRSSC, all participating physicians have specialized academic backgrounds in sleep medicine. Anaesthesiologists have evolved from primarily intraoperative specialists to comprehensive perioperative clinicians. This expanded role, aligned with the perioperative surgical home (PSH) model, enables anaesthesiologists to enhance safety and cost-effectiveness throughout the perioperative period.

DISE is increasingly used to select patients who may benefit from upper airway surgery for sleep-related breathing disorders [5]. In fact, it provides three-dimensional visualization of the upper airway during induced sleep, allowing for precise identification of obstruction sites. This procedure requires sedation to simulate natural sleep conditions, enabling dynamic observation of airway collapse. The implementation of a multidisciplinary-approved protocol for DISE marks a significant advancement in managing OSA patients. This protocol, developed collaboratively by the HRSSC ensures consistency and accuracy in assessing high-risk surgical candidates. Figure 1 illustrates the comprehensive, anaesthesiologist-led HRSSC model for the management of high-risk surgical OSA patients. By standardizing pre-procedural assessments, drug administration, endoscopic techniques, and post-procedural care, the protocol enhances diagnostic precision, facilitates collaborative decision-making, and optimizes surgical planning. The DISE procedure exemplifies a typical multidisciplinary approach, involving several steps such as administering sedation via a target-controlled infusion (TCI) system, examining the upper airways with a trans-nasal flexible fibre endoscope to classify obstruction sites, and discussing the procedure’s results and potential treatment options with the patient.

In conclusion, our anaesthesiology department is leading the HRSSC initiative, which aligns with the PSH model by focusing on preoperative assessment and comprehensive perioperative care. The HRSSC’s multidisciplinary approach ensures the best possible outcomes for high-risk OSA patients, promoting patient-centered care and advancing the quality of healthcare. The next step is to evaluate the effectiveness of this model in improving patient outcomes, reducing healthcare costs, and enhancing the overall quality of care.

## Figures and Tables

**Fig. 1:**
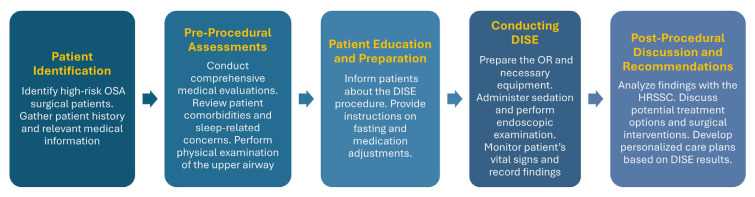
Multidisciplinary model for evaluating high-risk OSA surgical patients This flowchart represents the step-by-step process involved in the management of high-risk OSA surgical patients. It details the sequence from patient identification through pre-procedural assessments, patient education, the Drug-Induced Sleep Endoscopy (DISE) procedure, and the post-procedural discussions and recommendations. The model emphasizes the integration of multidisciplinary expertise to enhance patient outcomes and ensure comprehensive care planning.

## References

[1] Cooper Z Sayal P Abbett SK Neuman MD Rickerson EM Bader AM A conceptual framework for appropriateness in surgical care: reviewing past approaches and looking ahead to patient-centered shared decision making Anesthesiology 2015 123 1450 4 10.1097/ALN.0000000000000899 26495980 PMC4772867

[2] Kuiper BI Janssen LMJ Versteeg KS ten Tusscher BL van der Spoel JI Lubbers WD Does preoperative-multidisciplinary team assessment of high-risk patients improve the safety and outcomes of patients undergoing surgery? BMC Anesthesiol 2024 24 9 10.1186/s12871-023-02394-5 38166642 PMC10759340

[3] The Royal College of Anaesthetists Perioperative medicine: the pathway to better surgical care 2015 https://www.rcoa.ac.uk/sites/default/files/documents/2019-08/Perioperative%20Medicine%20-%20The%20Pathway%20to%20Better%20Care.pdf

[4] Kain ZN Fitch JCK Kirsch JR Mets B Pearl RG Future of anesthesiology is perioperative medicine: a call for action Anesthesiology 2015 122 1192 5 10.1097/ALN.0000000000000680 25886775

[5] Carrasco-Llatas M Matarredona-Quiles S De Vito A Chong KB Vicini C Drug-induced sleep endoscopy: technique, indications, tips and pitfalls Healthc Basel Switz 2019 7 93 10.3390/healthcare7030093 PMC6787696 31344900

